# Repetitive Peripheral Magnetic Stimulation Combined with Motor Imagery Changes Resting-State EEG Activity: A Randomized Controlled Trial

**DOI:** 10.3390/brainsci12111548

**Published:** 2022-11-15

**Authors:** Shun Sawai, Shoya Fujikawa, Ryu Ushio, Kosuke Tamura, Chihiro Ohsumi, Ryosuke Yamamoto, Shin Murata, Hideki Nakano

**Affiliations:** 1 Graduate School of Health Sciences, Kyoto Tachibana University, Kyoto 607-8175, Japan; 2 Department of Rehabilitation, Kyoto Kuno Hospital, Kyoto 605-0981, Japan; 3 Department of Physical Therapy, Faculty of Health Sciences, Kyoto Tachibana University, Kyoto 607-8175, Japan; 4 Department of Rehabilitation, Tesseikai Neurosurgical Hospital, Osaka 575-8511, Japan

**Keywords:** motor imagery, repetitive peripheral magnetic stimulation, combination, EEG, vividness

## Abstract

Repetitive peripheral magnetic stimulation is a novel non-invasive technique for applying repetitive magnetic stimulation to the peripheral nerves and muscles. Contrarily, a person imagines that he/she is exercising during motor imagery. Resting-state electroencephalography can evaluate the ability of motor imagery; however, the effects of motor imagery and repetitive peripheral magnetic stimulation on resting-state electroencephalography are unknown. We examined the effects of motor imagery and repetitive peripheral magnetic stimulation on the vividness of motor imagery and resting-state electroencephalography. The participants were divided into a motor imagery group and motor imagery and repetitive peripheral magnetic stimulation group. They performed 60 motor imagery tasks involving wrist dorsiflexion movement. In the motor imagery and repetitive peripheral magnetic stimulation group, we applied repetitive peripheral magnetic stimulation to the extensor carpi radialis longus muscle during motor imagery. We measured the vividness of motor imagery and resting-state electroencephalography before and after the task. Both groups displayed a significant increase in the vividness of motor imagery. The motor imagery and repetitive peripheral magnetic stimulation group exhibited increased β activity in the anterior cingulate cortex by source localization for electroencephalography. Hence, combined motor imagery and repetitive peripheral magnetic stimulation changes the resting-state electroencephalography activity and may promote motor imagery.

## 1. Introduction

Repetitive peripheral magnetic stimulation (rPMS) is a novel non-invasive technique for the application of repetitive magnetic stimulation to the peripheral nerves and muscles [1]. rPMS enhances muscle strength [2], increases muscle activity [3], reduces muscle tone [4], improves sensory function, and reduces pain [5]. In addition, it affects the central nervous system. rPMS induces intracortical inhibition [6] and promotes corticomotor excitability [7]. Furthermore, researchers have examined the effects of rPMS on electroencephalography (EEG); it decreases the activity of μ-waves [8] and β-waves [9]. These findings have focused attention on rPMS as a novel neurorehabilitation technique for stroke [10,11].

Repetitive transcranial magnetic stimulation (rTMS) is a non-invasive brain stimulation method using magnetic stimulation similar to rPMS. rTMS uses magnetic stimulation that passes through the scalp to alter cortical activity [12]. In addition, rTMS induces synaptic plasticity, besides attracting attention as a neurorehabilitation technique [13]. Specifically, its effectiveness has been verified in dementia [14], stroke [15], and depression [16]. However, rTMS directly stimulates the brain; thus, it is considered a relative contraindication for patients with epilepsy or seizures [17]. In contrast, rPMS stimulates the periphery and can be applied during the absence of implants around the stimulation. Thus, rPMS and rTMS use similar magnetic stimulation with different stimulation sites. rPMS is a safer stimulation method with a wider range of indications.

Motor imagery (MI) is a cognitive process in which a person imagines that he/she is exercising without actually moving or tensing the muscles [18]. MI improves the motor accuracy, muscle power, and flexibility of the motor system [19] even if the person is not actually exercising. In addition, MI shares certain brain activity with motor execution [20,21] and promotes corticomotor excitability [22,23]. Particularly, the primary motor area, supplementary motor area, premotor area, parietal lobe, and cerebellum are related to MI and motor execution [20]. In addition, peripheral regions, such as the left medial intraparietal sulcus, are related to both MI and motor execution [24,25,26]. Conversely, MI activates the bilateral posterior superior parietal cortex/precuneus and a small zone in the left precentral sulcus at the level of the middle frontal gyrus, compared with motor execution [20]. Recently, researchers have not only examined brain regions activated by MI but also networks among multiple regions, thus revealing a relationship between MI and integrated functional networks, including the frontal and posterior parietal lobes [27,28]. Furthermore, MI reportedly alters the EEG, thereby decreasing μ and β wave activity [29]. Thus, MI is used in neurorehabilitation [30]. Both rPMS and MI affect the peripheral and central organizations.

Furthermore, the combination of rPMS and MI promotes corticomotor excitability, compared with the use of each technique alone [31,32]. However, the combination of rPMS and MI does not promote corticomotor excitability in the antagonist muscles [33], and the effect of this combination is still under investigation. Despite reports on the effects of combined rPMS and MI on corticomotor excitability, researchers have not explored the effects of the combination on resting-state EEG. Resting-state EEG can assess motor imagery ability [34,35]; thus, researchers may accurately assess MI ability by examining the effects of the combination of rPMS and MI on resting EEG. In addition, measuring resting-state EEG is used to determine the efficiency of the MI-based brain–computer interface [34,35]. Thus, evaluating resting-state EEG is useful for clinical applications using the brain–computer interface.

Therefore, we aimed to clarify the effects of the combination of rPMS and MI on the vividness of MI and resting-state EEG. We hypothesized that the combination of MI and rPMS would increase the vividness of MI and modulate EEG activity, compared with MI alone. This is because the magnetic stimulation of rPMS may promote MI. By investigating the neural mechanisms underlying the combination of rPMS and MI, we can examine the effects of this novel intervention on brain function and neuroplasticity as well as its adaptation. This study will contribute to the evidence base for the combination of rPMS and MI.

## 2. Materials and Methods

### 2.1. Participants

We recruited 17 healthy young adults (20.71 ± 0.85 years) for the study. An assessment using the Edinburgh Handedness Inventory suggested all participants were right-handed [36]. We excluded the following participants: (i) with Mini-Mental State Examination [37] scores < 24, (ii) with orthopedic, neurological, or psychiatric diseases that may have affected the outcomes, (iii) with impaired right upper extremity movement, and (iv) with metal implants in the right upper extremity. The sample size was determined as 16 using the G*Power software [38] by considering an impact size of 0.40, α = 0.05, and power (1 − β) = 0.80 at a confidence level of 95%. This study was conducted in accordance with the tenets of the Declaration of Helsinki, and informed consent was obtained from all participants. This study was approved by the local institutional ethics committee of the Kyoto Tachibana University (approval no. 21–47).

### 2.2. Study Protocol

**Figure 1:**
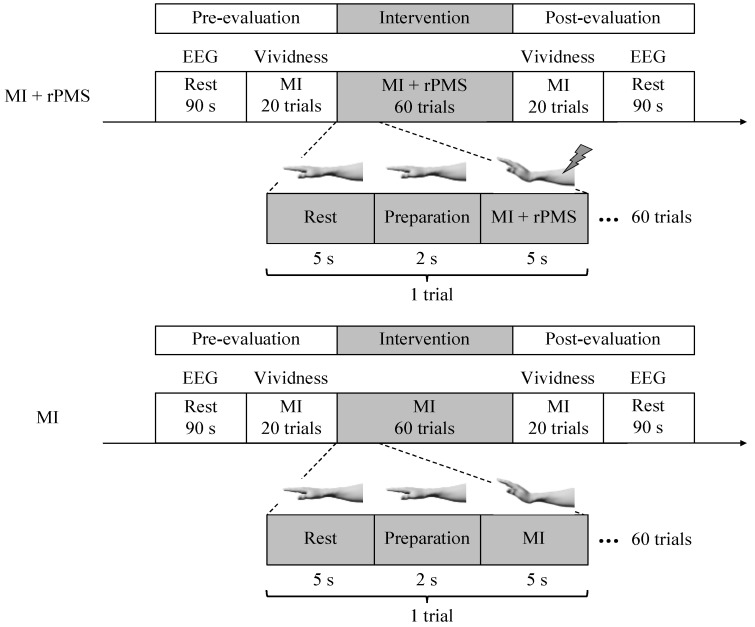
First, resting-state EEG is measured in the pre-evaluation phase. Subsequently, the participants perform the MI task 20 times, with rest (5 s), preparation (2 s), and MI (5 s) as one set. Subsequently, the vividness of MI is evaluated. In the intervention phase, the participants are randomly divided into the MI + rPMS group (*n* = 9) and MI group (*n* = 8). In the MI group, MI is performed 60 times using a procedure similar to that in the pre-evaluation phase. In the MI + rPMS group, we have applied rPMS to the right forearm during MI. Eventually, we have performed an evaluation in the post-evaluation phase, similar to that in the pre-evaluation phase. MI: motor imagery; rPMS: repetitive peripheral magnetic stimulation; and EEG: electroencephalography.

We performed a randomized controlled trial. First, resting-state EEG was measured for 90 s during the pre-evaluation phase. Subsequently, the participants performed rest (5 s), preparation (2 s), and MI (5 s) as a set for 20 times to evaluate the vividness of MI. In the intervention phase, the participants were randomly divided into the MI + rPMS group (*n* = 9) and MI group (*n* = 8). They were randomly assigned using random numbers generated by Microsoft Excel (Microsoft, Redmond, WA, USA). The randomization was conducted by the study investigators. The outcome assessor was blinded to the participant assignment. The MI group performed 60 MI sessions similar to that in the pre-evaluation phase. Conversely, we applied rPMS to the right forearm of those in the MI + rPMS group during MI. Eventually, we evaluated the vividness of MI and resting-state EEG in the post-evaluation phase, similar to that in the pre-evaluation phase (Figure 1).

### 2.3. Motor Imagery

In the intervention phase, the participants performed 60 repetitions of the MI task, which consisted of rest (5 s), preparation (2 s), and MI (5 s) as a set. The participants imagined a dorsiflexion movement of the right wrist joint. We used first-person, kinesthetic imagery for this experiment. The participants gazed at a viewpoint displayed on a monitor placed in front.

### 2.4. rPMS

**Figure 2:**
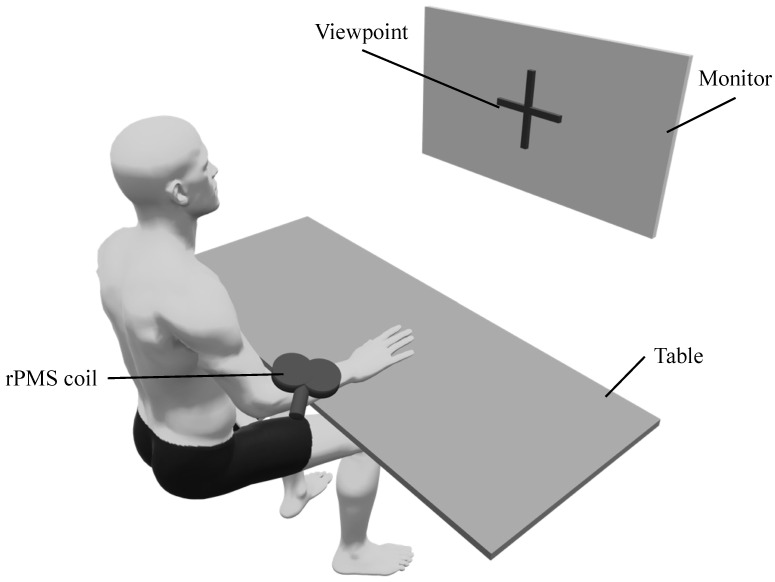
The environment of the intervention (MI + rPMS group). Both groups have performed the MI task, during which the participants gazed at a viewpoint displayed on a monitor. The MI task involves placing the right hand on the table and imagining a dorsiflexion movement of the wrist joint. In the MI + rPMS group, we have provided rPMS to the right extensor carpi radialis longus. rPMS induces dorsiflexion of the wrist during MI in the MI + rPMS group. rPMS: repetitive peripheral magnetic stimulation; MI: motor imagery.

Participants in the MI + rPMS group were provided with rPMS on the right forearm during the MI task. rPMS targeted the right extensor carpi radialis longus, and the motor points were identified by repetitive stimulation to determine the location and orientation of the PMS coil during MI. We placed the coil on the right extensor carpi radialis longus with the handle facing the proximal part (i.e., the upper arm) at an angle of 45° to the forearm (Figure 2). rPMS was provided using a magnetic stimulation machine (MagPro R20; MagVenture, Inc., Farum, Denmark) and an rPMS coil (MC-B70; MagVenture, Inc., Farum, Denmark). The rPMS coil used in this study was a figure-eight coil. The inner and outer diameters of this coil are 27 mm and 97 mm, respectively, with a pulse width of 280 μs. The stimulation frequency, stimulation duration, and stimulation intensity was set to 20 Hz [39], 5 s, and 1.2 times the motor threshold [3], respectively. The motor threshold was the minimum intensity at which the muscle contracted upon stimulation and was visually assessed by two examiners [32]. rPMS induced dorsiflexion of the wrist joint during stimulation.

### 2.5. Measures

We evaluated the effects of MI and rPMS by assessing the vividness of MI and resting-state EEG before and after the intervention phase (Figure 1). We assessed the vividness of MI [40] with an MI task, similar to that in the intervention phase. The subjective vividness of MI was evaluated using the Visual Analog Scale (VAS). The MI task in the evaluation phase did not provide rPMS for both the MI and MI + rPMS groups.

We measured the resting-state EEG under closed-eye conditions using an electroencephalograph (MP-6100; Miyuki Giken, Co., Ltd., Tokyo, Japan) and active dry electrodes (Miyuki Giken, Co., Ltd., Tokyo, Japan), Tokyo, Japan). The EEG was recorded on 19 channels (Fp1, Fp2, F7, F3, Fz, F4, F8, T3, C3, Cz, C4, T4, T5, P3, Pz, P4, T6, O1, and O2) in accordance with the international 10–20 method; the reference electrode and earth electrode were installed in the left earlobe. The recorded EEG was downsampled to 512 Hz using EEGLAB in MATLAB (Mathworks, Inc., Natick, MA, USA), and the bandpass filter was set from 1 Hz to 40 Hz. Subsequently, we performed an independent component analysis to remove artifacts, such as the heartbeat, muscle activity, and channel noise from the EEG. We used exact low-resolution brain electromagnetic tomography (eLORETA) to reconstruct cortical current density distributions from the processed EEG. eLORETA used the Montreal Neurological Institute 152 template. It consists of 6239 cortical voxels with a spatial resolution of 5 mm in a realistic head model. In eLORETA, the coordinates of the 19 electrodes were fitted to a probabilistic anatomical template of the Talairach atlas. These coordinates were used to compute the eLORETA transformation matrix. Following transformation to the average standard EEG activity, we averaged the artifact-free 1-s epochs and calculated the cross-spectra were calculated in eLORETA for the β-wave frequency band of each participant. β-wave modulation occurred in both rPMS [9] and MI [29]; thus, we analyzed the β-wave activity.

### 2.6. Statistical Analysis

First, we confirmed the normality of the VAS using the Shapiro–Wilk test. Consequently, we performed a two-way analysis of variance (ANOVA) with two factors, namely group (MI group, MI + rPMS group) and time (pre, post). A post hoc Bonferroni test was conducted to determine the group or periods that displayed significant differences. This analysis compared VAS scores before and after the intervention. We used SPSS ver. 28.0 (IBM Corp., Armonk, NY, USA) for the statistical analysis.

eLORETA was used for a comparison of the resting-state EEG before and after the intervention and an assessment of group differences. eLORETA log-transformed current power [41] was used to perform F-tests corresponding to each voxel in the beta frequency band. In the 3-D images obtained by statistical analysis, voxels displaying significant differences were detected by statistical non-parametric mapping. The statistical significance level was set at *p* < 0.05 for all analyses.

## 3. Results

**Figure 3:**
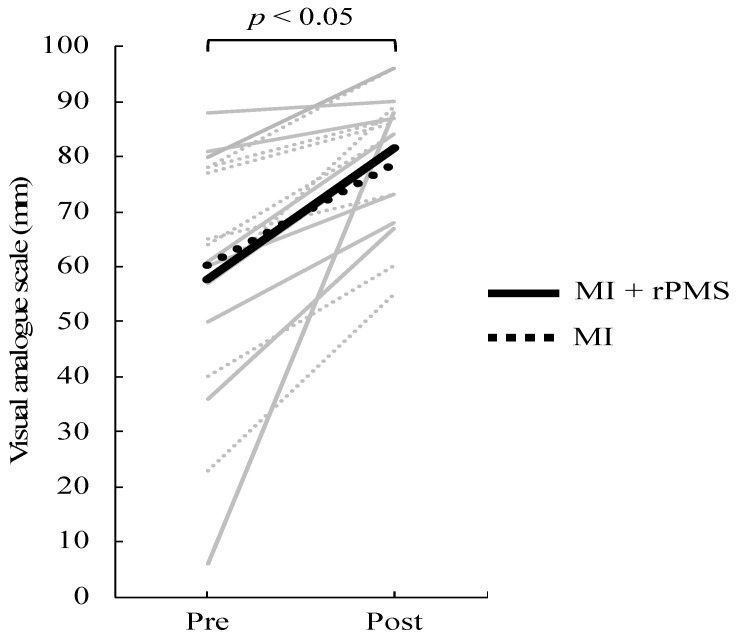
Change in VAS scores before and after the intervention. The solid line depicts data for the MI + rPMS group, whereas the dotted line depicts data for the MI group. Gray indicates data for each participant, and black indicates the mean value for each group. VAS scores have increased significantly following the intervention in both the MI + rPMS and MI groups (*p* < 0.01). MI: motor imagery; rPMS: repetitive peripheral magnetic stimulation; and VAS: Visual Analog Scale.

**Figure 4:**
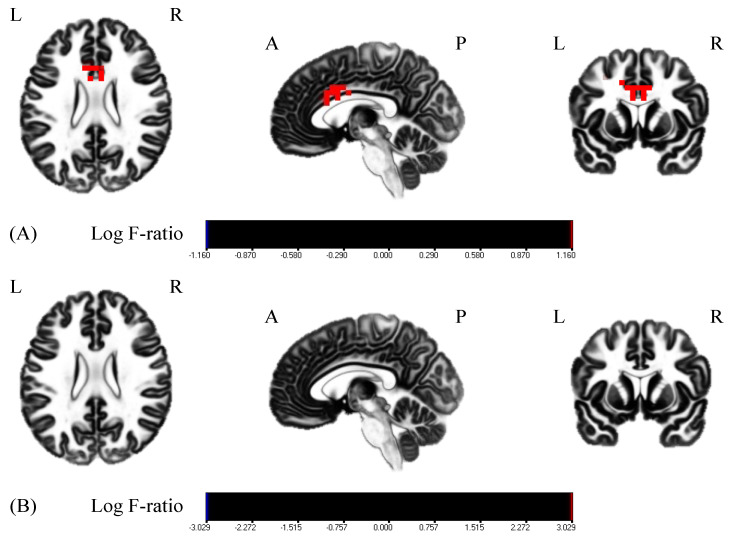
Comparisons of the resting-state EEG before and after the intervention. Red areas indicate the areas of increased activity following the intervention. (**A**) EEG comparisons in the MI + rPMS group. ACC β activity has increased significantly following the intervention (*p* < 0.05). (**B**) EEG comparisons in the MI group. There is no change in β activity in any brain region before and after the intervention (*p* > 0.05). MI: motor imagery; rPMS: repetitive peripheral magnetic stimulation; EEG: electroencephalography; and ACC, anterior cingulate cortex.

Results are presented as means ± standard deviation (SD). First, VAS scores increased from 60.25 ± 19.84 to 78.75 ± 14.64 and from 57.67 ± 25.44 to 81.56 ± 10.16 in the MI group and MI + rPMS group, respectively. The two-way ANOVA revealed a significant main effect with the time factor (F = 22.37, *p* < 0.01) (Figure 3). The MI + rPMS group demonstrated a significant increase in the β activity of the anterior cingulate cortex (ACC) following the intervention (*p* < 0.05) (Figure 4, Table 1). In contrast, no brain regions in the MI group demonstrated significant changes in the activity before and after the intervention (*p* > 0.05) (Figure 4). However, EEG comparisons revealed no significant differences between the MI and MI + rPMS groups following the intervention.

## 4. Discussion

We aimed to investigate the effects of combined rPMS and MI on the vividness of MI and resting-state EEG. Both MI alone and the combination of rPMS and MI significantly increased the vividness of MI following the intervention, despite no significant between-group differences. In addition, resting-state EEG comparisons revealed that the combination of rPMS and MI increased ACC β activity.

The vividness of MI increased following the intervention in both the MI and MI + rPMS groups. However, there was no significant main effect difference between the groups; VAS-based assessment of the vividness of MI is reportedly associated with neural activity [42,43]. In the present study, both the MI and MI + rPMS groups demonstrated increased vividness of MI following the intervention. Repeated MI increases the vividness of MI [44]. In the present study, both groups underwent repeated MI, thereby increasing the vividness of MI. However, there was no difference in the vividness of MI between the groups. In other words, rPMS may not promote MI in terms of subjective evaluation. A previous study that examined the effects of repeated MI on the vividness of MI using VAS reported that VAS scores improved from 60 mm (before) to 80 mm (after) [44]. In addition, a previous study examining the effects of MI combined with action observation on the vividness of MI revealed improvement in VAS scores from 50 mm (before) to 80 mm (after) [40]. Similarly, the VAS score improved from 60 mm (before) to 80 mm (after) in both groups in the present study. Taken together, there were no differences in the VAS scores with MI alone and upon adding action observation or rPMS to MI. Therefore, additional interventions may marginally affect the repetition of MI during the subjective assessment.

The MI group did not demonstrate changes in the resting-state EEG before and after the intervention, whereas the MI + rPMS group demonstrated an increase in ACC β activity. ACC is associated with error detection [45], cognitive control [46], and attention functions [47,48]. Particularly, ACC is not only associated with error detection but also with feedback [49]. Moreover, this process promotes motor learning [50]. In this study, rPMS generated passive wrist dorsiflexion movement. Moreover, it was possible to detect errors based on the muscular sensory information from the imagined and muscular sensory input from the actual movement. This phenomenon may have induced a feedback signal to modify the error, thus resulting in an increased ACC activity. In addition, ACC is involved in the construction and updating of internal models [51]. Internal model updating is important in MI for predicting the sensations induced by imagined movements [52,53]. In this study, applying rPMS to MI increased the ACC activity. rPMS-generated passive wrist dorsiflexion movements may have increased ACC activity by providing input myosensory information and updating the internal model. In addition, cognitive control [54,55] and attentional function [56] are associated with MI; the ACC plays an important role in these functions [46,47,48]. In this study, ACC activity increased following the intervention only in the MI + rPMS group, thus suggesting rPMS may improve attentional and cognitive functions during MI and increase the ACC activity. However, there was no significant difference in the post-intervention EEG between the groups. The eLORETA structure did not facilitate this verification; nonetheless, the MI group’s EEG supposedly displayed variability than that of the MI + rPMS group. MI comprises two modalities, namely kinesthetic imagery and visual imagery. Moreover, the characteristics of MI vary among individuals [57,58,59]. Kinesthetic and visual modalities exhibit different EEG [60]. Therefore, the effects of MI vary among individuals. Moreover, rPMS may have eliminated these differences. In other words, rPMS may eliminate the variability in the effects of MI and change ACC activity related to cognition and attention.

## 5. Limitations and Future Directions

This study had several limitations. First, we did not evaluate the personality traits that may have influenced the ability to attribute mental states. An evaluation of personality traits would have enabled an accurate examination of the effects of interventions. Moreover, it would enable considering the adaptation of the intervention. Second, we performed only subjective evaluation of MI; both subjective and objective evaluations of MI may provide details of the effect of rPMS on MI. Third, we measured the resting-state EEG and not EEG during the task. rPMS affects EEG; thus, we used resting-state EEG rather than the task EEG. In the present study, resting-state EEG reflected the combined effects of MI and rPMS. However, EEG during the task may account for different brain oscillatory changes and their effects on MI. In addition, future studies can examine retention and clarify its effects on motor learning to obtain clinically relevant results.

## 6. Conclusions

In this study, both MI and MI + rPMS groups significantly improved the vividness of MI. In addition, the MI + rPMS group revealed increased ACC β activity following the intervention. The ACC is involved in error detection and internal model updating; this function promotes motor learning. rPMS generated sensory input to MI, and ACC activity may have increased during error modification between MI and the actual movement. The impact of MI + rPMS on the resting-state EEG will reveal the effects and mechanisms of this intervention. Thus, this study will likely contribute to devising novel interventions that combine MI and rPMS to promote MI.

## Figures and Tables

**Table 1:** Brain regions with significantly increased beta activity following the intervention.

Group	Brain Region	BA	MNI Coordinates	*p*-Value
			(x, y, z)	
MI + rPMS	Anterior cingulate cortex	24	(−5, 10, 30)	*p* < 0.05
		32	(−15, 15, 35)	
		33	(−5, 10, 25)	

This table indicates brain regions with significantly increased β activity following the intervention. The MI + rPMS group displayed increased β activity of the anterior cingulate cortex, compared with the MI group. MI: motor imagery; rPMS: repetitive peripheral magnetic stimulation; BA: brodmann area; and MNI: Montreal Neurological Institute.

## Data Availability

The data used to support the findings of this study shall be made available from the corresponding author upon request. The data are not publicly available because they contain information that can compromise the privacy of the research participants.
